# Supplementary material for: Genome-Wide Association Study of Body Conformation Traits by Whole Genome Sequencing in Dazu Black Goats
Source: Animals (Basel). 2022 Feb 23;12(5):548. doi: 10.3390/ani12050548 (PMC8908837; doi:10.3390/ani12050548)
Supplement: Supplementary file 1 [file animals-12-00548-s001.zip › Figure S1 The National Standard for the body measurements of Goats in China.pdf]

Body height:
The vertical distance from the highest point of the scapula to the ground when the measured goat stands upright on a firm and flat surface Measurements were taken in cm and results were retained to one decimal place. Measurements were made with a calipers.

Body length:
The distance from the Sternum to the posterior end of the sciatic tuberosity in a straight line when the measured goat stands upright on a firm and flat surface. Measurements were taken in cm and results were retained to one decimal place. Measurements were made with a calipers.

Cannon circumference:
The horizontal circumference of the thinnest part of the left forelimb tuberosity when the measured goat stands upright on a firm and flat surface. Measurements were taken in cm and results were retained to one decimal place.Measurements were made with a tape measure.

Chest depth:
The vertical distance from the highest point of the scapula to the Sternum when the measured goat stands upright on a firm and flat surface. Measurements were taken in cm and results were retained to one decimal place.Measurements were made with a calipers.

Chest width:
The straight line distance between the left and right sides of the widest part of the scapula when the measured goat stands upright on a firm and flat surface. Measurements were taken in cm and results were retained to one decimal place.Measurements were made with a calipers.

Heart girth:
The length of the posterior end of the scapula around the chest when the measured goat stands upright on a firm and flat surface. Measurements were taken in cm and results were retained to one decimal place.Measurements were made with a tape measure.
