# Supplementary figures and images for: Genome-Wide Association Study of Body Conformation Traits by Whole Genome Sequencing in Dazu Black Goats
Source: Animals (Basel). 2022 Feb 23;12(5):548. doi: 10.3390/ani12050548 (PMC8908837; doi:10.3390/ani12050548)

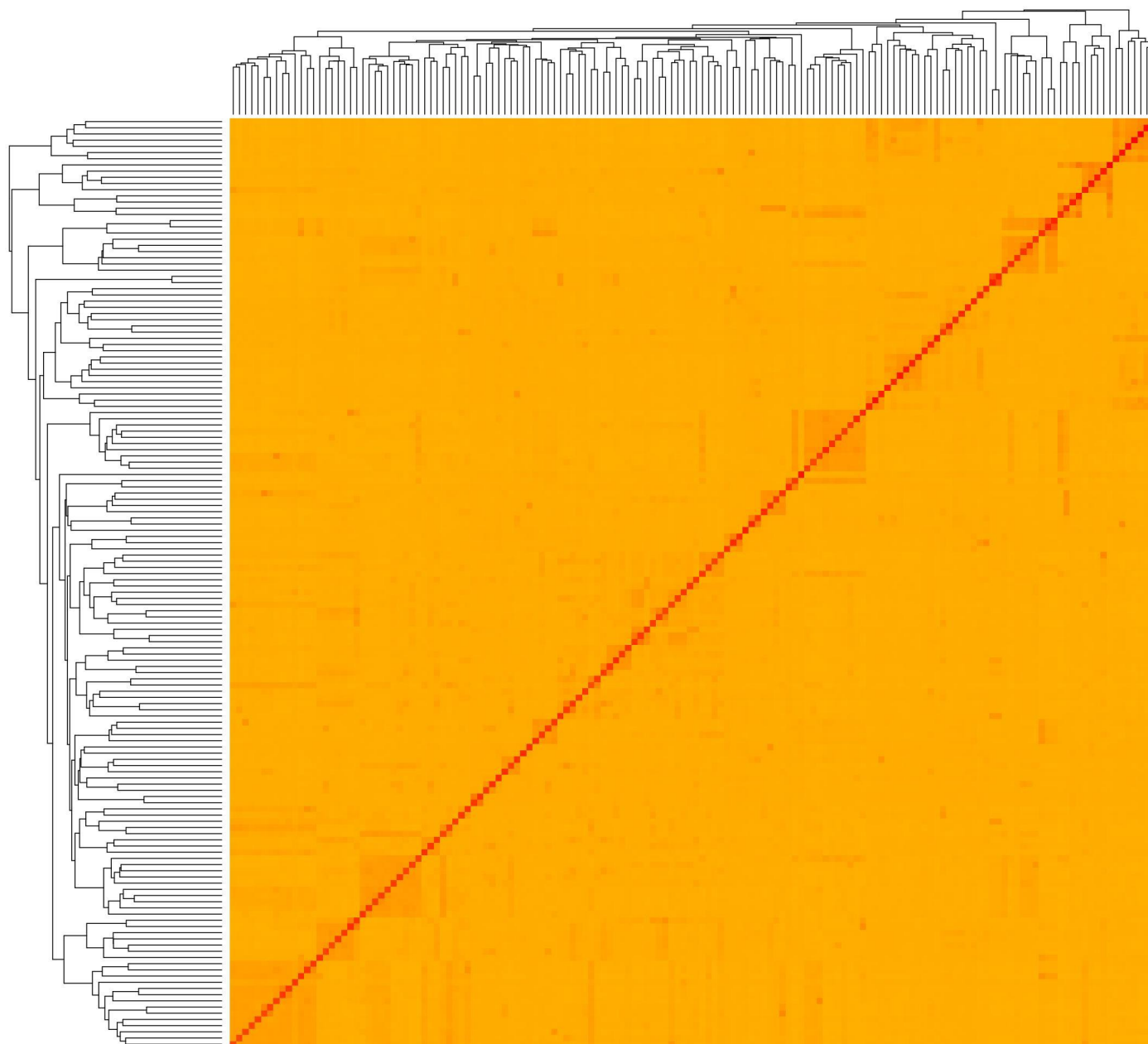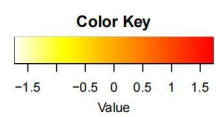

Supplement: Supplementary file 1 [file animals-12-00548-s001.zip › Figure S3 kinship relationships.pdf]
